# A novel bifunctional GH51 exo-α-l-arabinofuranosidase/endo-xylanase from *Alicyclobacillus* sp. A4 with significant biomass-degrading capacity

**DOI:** 10.1186/s13068-015-0366-0

**Published:** 2015-11-30

**Authors:** Wenxia Yang, Yingguo Bai, Peilong Yang, Huiying Luo, Huoqing Huang, Kun Meng, Pengjun Shi, Yaru Wang, Bin Yao

**Affiliations:** Key Laboratory for Feed Biotechnology of the Ministry of Agriculture, Feed Research Institute, Chinese Academy of Agricultural Sciences, No. 12 Zhongguancun South Street, Beijing, 100081 People’s Republic of China

**Keywords:** *Alicyclobacillus* sp. A4, α-l-Arabinofuranosidase (Abf), Xylanase, Glycosyl hydrolase (GH), Synergism

## Abstract

**Background:**

Improving the hydrolytic performance of xylanolytic enzymes on arabinoxylan is of importance in the ethanol fermentation industry. Supplementation of debranching (arabinofuranosidase) and depolymerizing (xylanase) enzymes is a way to address the problem. In the present study, we identified a bifunctional α-l-arabinofuranosidase/endo-xylanase (Ac-Abf51A) of glycoside hydrolase family 51 in *Alicyclobacillus* sp. strain A4. Its biochemical stability and great hydrolysis efficiency against complex biomass make it a potential candidate for the production of biofuels.

**Results:**

The gene encoding Ac-Abf51A was cloned. The comparison of its sequence with reference proteins having resolved 3D-structures revealed nine key residues involved in catalysis and substrate-binding interaction. Recombinant Ac-Abf51A produced in *Escherichia coli* showed optimal activity at pH 6.0 and 60 °C with 4-nitrophenyl-α-l-arabinofuranoside as the substrate. The enzyme exhibited an exo-type mode of action on polyarabinosides by catalyzing the cleavage of α-1,2- and α-1,3-linked arabinofuranose side chains in sugar beet arabinan and water-soluble wheat arabinoxylan and α-1,5-linked arabinofuranosidic bonds in debranched sugar beet arabinan. Surprisingly, it had capacity to release xylobiose and xylotriose from wheat arabinoxylan and was active on xylooligosaccharides (xylohexaose 1.2/mM/min, xylopentaose 6.9/mM/min, and xylotetraose 19.7/mM/min), however a lower level of activity. Moreover, Ac-Abf51A showed greater synergistic effect in combination with xylanase (2.92-fold) on wheat arabinoxylan degradation than other reported enzymes, for the amounts of arabinose, xylose, and xylobiose were all increased in comparison to that by the enzymes acting individually.

**Conclusions:**

This study for the first time reports a GH51 enzyme with both exo-α-l-arabinofuranosidase and endo-xylanase activities. It was stable over a broad pH range and at high temperature, and showed greater synergistic effect with xylanase on the degradation of wheat arabinoxylan than other counterparts. The distinguished synergy might be ascribed to its bifunctional α-l-arabinofuranosidase/xylanase activity, which may represent a possible way to degrade biomass at lower enzyme loadings.

**Electronic supplementary material:**

The online version of this article (doi:10.1186/s13068-015-0366-0) contains supplementary material, which is available to authorized users.

## Background

Hemicellulose, the second most abundant polysaccharide in plants, combines with cellulose and lignin to compose lignocellulose of plant cell walls, and accounts for about 20–35 % of lignocellulosic biomass [1, 2]. It mainly consists of xylan, glucuronoxylan, arabinoxylan, glucomannan, and xyloglucan, and has attracted much attention for their industrial importance in bioconversion of plant biomass to biofuel, improvement of animal feedstock digestibility, and organic synthesis [3, 4]. Heterogeneous xylans are the major constituents of hemicellulose. Among them, arabinoxylans such as those found in wheat straw [5] consist of a backbone of β-1,4-linked d-xylopyranose residues that are extensively decorated at C-2 and/or C-3 positions with arabinofuranose side chains [6, 7]. Arabinan, a component of pectin, contains a backbone of α-1,5-linked l-arabinofuranosyl residues as well as α-1,2- and α-1,3-linked side chains [8]. Thus, due to the structural complexity of xylans, efficient hydrolysis of wheat arabinoxylan to achieve high xylose yields by complete xylan monomerization requires supplementing xylanases with α-l-arabinofuranosidases and other accessory enzymes [9].

Exo-acting α-l-arabinofuranosidases (Abfs, EC 3.2.1.55) catalyze the hydrolysis of terminal non-reducing α-1,2-, α-1,3-, and α-1,5-l-arabinofuranosyl residues [10]. In recent years, Abfs have received much attention because of their potential applications in the processing of fruits and cereals for aroma improvement and the conversion of hemicellulose to fuels and chemicals [8, 11–13]. Based on amino acid sequence, primary structure similarity, and hydrophobic cluster analysis, Abfs have been classified into glycosyl hydrolase (GH) families 3, 43, 51, 54, and 62 [14, 15]. Members of different families display specific preference for substrates. For example, those of GH43 hydrolyze α-1,5-linked arabinofurano-oligosaccharides, GH62 Abfs show absolute specificity for arabinoxylan, and members of GH51 and GH54 catalyze the removal of both α-1,2 and α-1,3-linked arabinofuranose side chains from arabinan and xylan [16].

Until now, bacterial GH51 Abfs from *Anoxybacillus kestanbolensis* [17], *Clostridium stercorarium* [18], *Geobacillus stearothermophilus* [19], *Bacillus pumilus* [20], *Bifidobacterium longum* [21], and *Streptomyces* spp. [22–24] have been characterized, and the structures of six bacterial Abfs have been resolved, which all perform hydrolysis via a retaining mechanism [25]. Previous studies indicate that GH51 Abfs have various modes of action on different substrates. This broad specificity against distinct branching modifications represents a great advantage for biotechnological processing of complex and branched polysaccharides [26]. In this study, we report the characterization of a novel *Alicyclobacillus* sp. A4 GH51 α-l-arabinofuranosidase (Ac-Abf51A) with both exo-α-l-arabinofuranosidase and endo-xylanase activities, which is significantly different from previously reported GH51 arabinofuranosidases in substrate specificity and would play an important role in biomass hydrolysis.

## Results

### Gene cloning and sequence analysis

**Fig. 1:**
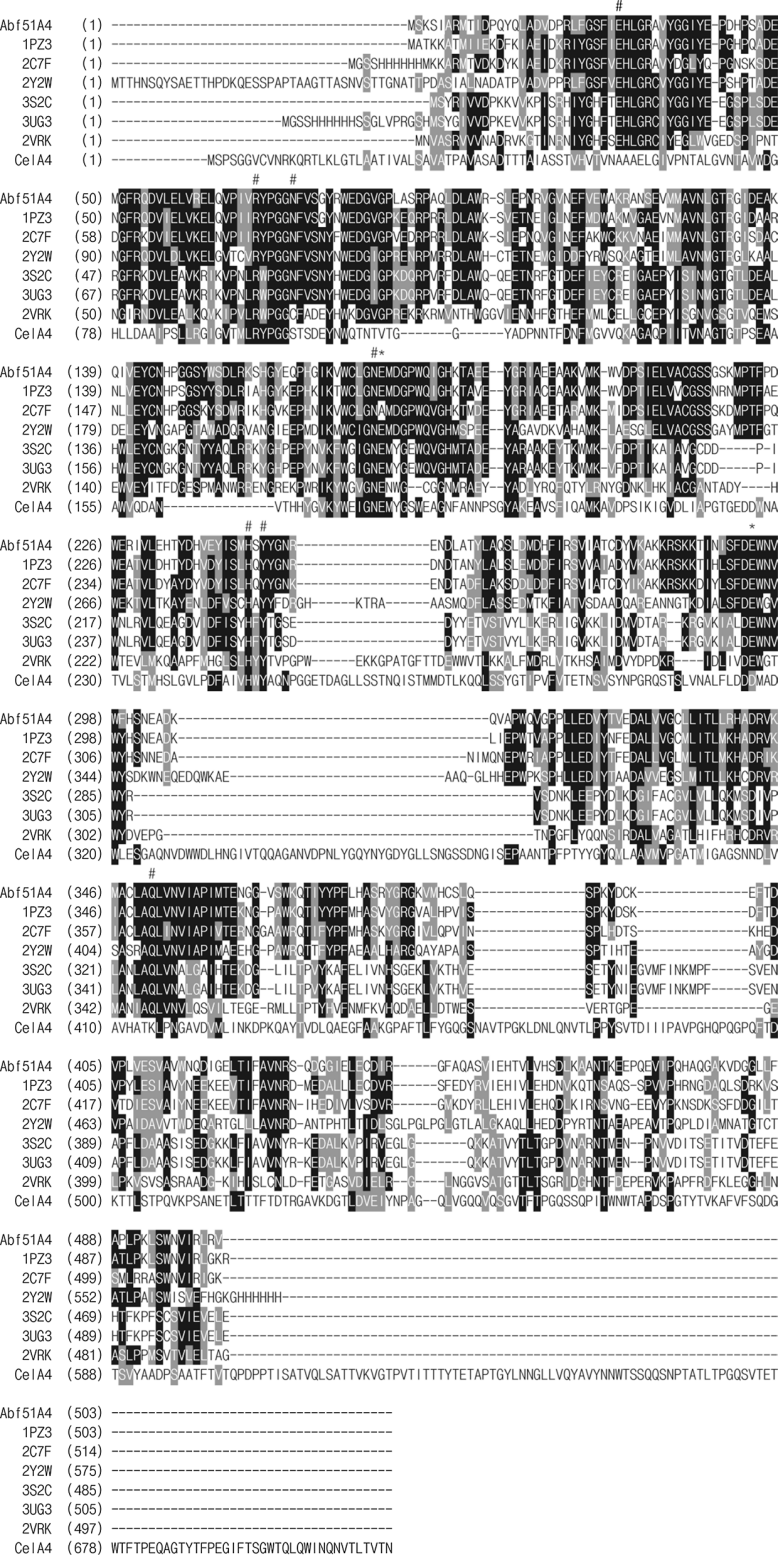
Amino acid sequence alignment of Ac-Abf51A from *Alicyclobacillus* sp. A4 with CelA4 from *Alicyclobacillus* sp. A4 (ADI82825.1) and other GH51 Abfs from *Bifidobacterium longum* B667 (PDB: 2Y2 W), *Geobacillus stearothermophilus* T6 (PDB: 1PZ3), *Clostridium thermocellum* ATCC 27405 (PDB: 2C7F), *Thermobacillus xylanilyticus* D3 (PDB: 2VRK), *Thermotoga maritima* MSB8 (PDB: 3UG3), and *Thermotoga petrophila* RKU-1 (PDB: 3S2C), using the ClustalW program. Identical and similar amino acids are indicated by *black and gray shades*, respectively. The catalytic glutamate residues are indicated by *asterisk*, and the conserved residues are indicated by *hash*

The full-length Abf gene, *Ac*-*abf51A* (GenBank accession no.KT781102), contains 1509 bp and encodes a 502-residue polypeptide with a calculated molecular mass of 56.7 kDa. The deduced amino acid sequence of Ac-Abf51A is most similar to a putative Abf of *Alicyclobacillus hesperidum* (99 % identity; WP_006446014.1), and 68 % identical with the crystal structure-resolved Abf from *G. stearothermophilus* T6 (1PZ3). Using the Accelrys Discovery Studio software with 1PZ3 as the template, modeled Ac-Abf51A folds into two modules: the N-terminal catalytic module of the frequently encountered (α/β)_8_ barrel (TIM barrel) of GH51 and the C-terminal module of 12-stranded β-sandwich with a jelly-roll topology (Additional file 1). The structure of the (α/β)_8_ barrel domain places Ac-Abf51A into the superfamily of clan GH-A, in which two conserved glutamates (Glu175 and Glu294) were found on strands β-4 (acid/base) and β-7 (nucleophile), respectively (Fig. 1). Based on homology analysis of amino acid sequences with the characterized GH51 Abf with crystal structure [17], nine key residues including Glu29, Arg69, Asn74, Asn174, Glu175, His244, Tyr246, Glu294, and Gln351 are conserved and probably involved in catalysis and substrate-binding interaction (Fig. 1).

### Expression and purification of recombinant Ac-Abf51A

**Fig. 2:**
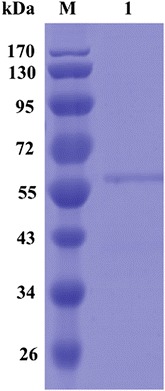
SDS-PAGE analysis of the purified recombinant Ac-Abf51A. Lanes: M, the standard protein molecular weight markers; 1, the recombinant Ac-Abf51A purified by Ni^2+^-NTA affinity chromatography

After induction with IPTG at 30 °C for 6 h, the whole *E. coli* cell lysates showed an Abf activity of 2.6 U/mL. The recombinant Ac-Abf51A was purified by one-step immobilized metal-affinity chromatography (IMAC). The specific activity of purified Ac-Abf51A was 18.2 U/mg with 4-nitrophenyl-α-l-arabinofuranoside as the substrate. The purified enzyme migrated as a single band with an apparent molecular mass of 57.0 kDa, as determined by SDS-PAGE analysis (Fig. 2).

### Biochemical characterization

**Fig. 3:**
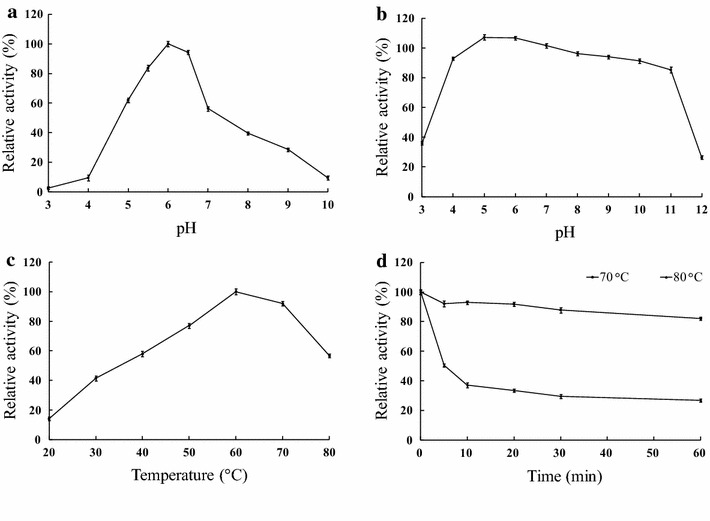
Characterization of the purified recombinant Ac-Abf51A with 4-nitrophenyl-α-L-arabinofuranoside as the substrate. **a** The effect of pH on enzyme activity. The activity assays were performed at 60 °C in buffers of pH 3.0–10.0 for 10 min. **b** pH stability of Ac-Abf51A. After pre-incubating the enzyme at 37 °C for 1 h at pH 3.0–12.0, the residual activities were measured in 100 mM McIlvaine buffer (pH 6.0, 60 °C and 10 min). **c** The effect of temperature on enzyme activity measured in 100 mM McIlvaine buffer (pH 6.0) for 10 min. **d** Thermostability of purified Ac-Abf51A. The enzyme was pre-incubated at 70 °C or 80 °C in 100 mM McIlvaine buffer (pH 6.0) for various periods, followed by activity assay at pH 6.0 and 60 °C for 10 min

With 4-nitrophenyl-α-l-arabinofuranoside as the substrate, Ac-Abf51A exhibited the highest Abf activity at pH 6.0 (Fig. 3a). The enzyme retained more than 80 % of the initial activity after incubation at pH 4.0–11.0, 37 °C for 1 h (Fig. 3b). When assayed at pH 6.0, Ac-Abf51A was most active at 60 °C (Fig. 3c). The enzyme retained full activity after 12-h incubation at 60 °C and 65 °C, more than 80 % of the initial activity at 70 °C for 1 h, and more than 30 % of the activity at 80 °C for 30 min (Fig. 3d).

Most metal ions and chemicals had little or no effect on Ac-Abf51A (Table 1). However, the presence of K^+^, Co^2+^, Ag^+^, and SDS strongly inhibited the enzymatic activity of Ac-Abf51A.

**Table 1:** Effect of metal ions and chemical reagents (5 mM) on the activity of purified recombinant Ac-Abf51A

Chemicals	Relative activity (%)^a^
None	100.0 ± 1.5
Mg^2+^	110.0 ± 1.8
Zn^2+^	108.5 ± 1.7
Na^+^	108.5 ± 1.7
Li^+^	103.0 ± 1.6
Cr^3+^	101.9 ± 1.4
Fe^3+^	100.7 ± 2.0
Cu^2+^	99.2 ± 0.7
Mn^2+^	98.0 ± 1.0
Ni^2+^	96.3 ± 1.2
Pb^2+^	86.7 ± 1.5
Ca^2+^	81.7 ± 2.6
K^+^	58.1 ± 1.1
Co^2+^	25.4 ± 0.5
Ag^+^	9.6 ± 1.3
β-Mercaptoethanol	93.2 ± 2.4
EDTA	92.1 ± 2.1
SDS	47.5 ± 0.6

^a^Values represent the mean ± SD (*n* = 3) relative to the untreated control samples

### Substrate specificity and kinetic parameters

Ac-Abf51A had broad substrate specificity. The capacity of purified Ac-Abf51A to hydrolyze a range of different substrates was evaluated. For 4-nitrophenyl-glycosides and polysaccharides with reaction time within 10 min, it acted solely on 4-nitrophenyl-α-l-arabinofuranoside. When the incubation period was lengthened to 5 h, Ac-Abf51A showed weak activity against 4-nitrophenyl-β-d-xylopyranoside (0.026 % activity relative to that on 4-nitrophenyl-α-l-arabinofuranoside). The *K*_m_, *V*_max_, and *k*_cat_ values of Ac-Abf51A towards 4-nitrophenyl-α-l-arabinofuranoside were determined to be 0.46 mM, 24.4 μmol/min/mg, and 23.0/s, respectively. The *k*_cat_/*K*_m_ value was 50/mM/s.

For longer incubation (up to 12 h), it was active on sugar beet arabinan and debranched sugar beet arabinan, releasing arabinose as the final product (0.57 and 0.30 mg/mL, respectively) (Additional file 2). It also released arabinose, xylobiose, xylotriose, and xylotetraose from water-soluble wheat arabinoxylan.

**Fig. 4:**
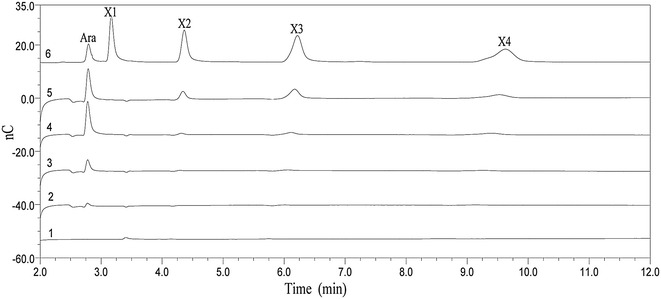
Time course of hydrolysis of soluble wheat arabinoxylan by Ac-Abf51A. 1, the control of substrate incubated without enzyme for 24 h; 2–5, the hydrolysate with enzyme treatment for 1 h, 6 h, 12 h, and 24 h, respectively; 6, the xylooligosaccharide standards: *Ara* arabinose, *X1* xylose, *X2* xylobiose, *X3* xylotriose, *X4* xylotetraose

Further time course analysis of the water-soluble wheat arabinoxylan digestion by Ac-Abf51A indicated that arabinose was yielded as the sole product at the beginning, and xylobiose, xylotriose, and xylotetraose were also released after a prolonged incubation (Fig. 4). When using xylooligosaccharides as the substrates, Ac-Abf51A exhibited an endo-mode of action on xylohexaose, xylopentaose and xylotetraose, releasing products of lower degree of polymerization. However, it showed barely detectable activity on xylotriose and xylobiose. The *k*_cat_/*K*_m_ values of Ac-Abf51A on xylotetraose, xylopentaose, and xylohexaose were 1.2/mM/min, 6.9/mM/min, and 19.7/mM/min, respectively (Additional file 3).

### Characterization of four mutants of Ac-Abf51A

Four Ac-Abf51A mutants containing the (α/β)_8_ barrel module alone (Ac-Abf51An), the jelly-roll module alone (Ac-Abf51Ac), and the two catalytic glutamic acid residues substituted by glutamine (E175Q and E194Q), respectively, were successfully expressed in *E. coli* BL21 (DE3) and purified to homogeneity by Ni–NTA columns individually. Except for Ac-Abf51An that has both exo-α-l-arabinofuranosidase and endo-xylanase activities, other three mutant proteins were completely inactive towards 4-nitrophenyl-α-l-arabinofuranoside, water-soluble wheat arabinoxylan, and xylotetraose, xylopentaose, and xylohexaose. The results showed that Ac-Abf51A catalyzes the two types of substrate at the same active sites within a single catalytic domain.

### Synergetic effects

The product composition of water-soluble wheat arabinoxylan hydrolysis by Ac-Abf51A and endo-xylanase XynBE18 individually or in combination were determined by HPAEC-PAD. XynBE18 released only xylose and xylobiose, and Ac-Abf51A liberated arabinose and trace amounts of xylobiose and xylotriose. In comparison with the hydrolysis products by XynBE18 or Ac-Abf51A alone, all enzyme combinations had significant synergistic effects on wheat arabinoxylan degradation, producing substantially more arabinose, xylobiose, and xylose. The greatest synergy showed no difference, when simultaneous incubation of wheat arabinoxylan with Ac-Abf51A and XynBE18 (2.92-fold increase, Table 2). First addition of Ac-Abf51A followed by XynBE18 had no difference with XynBE18 alone on the production of arabinose and xylose. However, the amount of xylobiose was increased by 1.4-fold (1.26-fold increase in total, Table 2). When XynBE18 was added followed by Ac-Abf51A, more arabinose, xylobiose, and xylotriose were released (2.12-fold increase, Table 2).

**Table 2:** Simultaneous and sequential reactions of Ac-Abf51A and XynBE18 against water-soluble wheat arabinoxylan

Enzyme added		Amounts of xylooligosaccharides (mg/mL)			Amounts of arabinose (mg/mL)	Degree of synergy^a^
First reaction	Second reaction	Xylose	Xylobiose	Xylotriose		
XynBE18	None	0.26 ± 0.01	0.45 ± 0.02	–	–	–
Ac-Abf51A	None	–	0.004 ± 0.00	0.006 ± 0.00	0.14 ± 0.01	–
Ac-Abf51A	XynBE18	0.30 ± 0.01	0.63 ± 0.03	–	0.14 ± 0.01	1.26
XynBE18	Ac-Abf51A	0.21 ± 0.01	0.61 ± 0.02	0.20 ± 0.01	0.77 ± 0.03	2.12
XynBE18 + Ac-Abf51A	None	0.62 ± 0.03	1.14 ± 0.04	–	0.72 ± 0.04	2.92

The amounts of xylooligosaccharides and arabinose were determined by HPAEC

–, not detected

^a^Degree of synergy is defined as the ratio of the amounts of saccharide released from simultaneous or sequential enzyme combinations to the sum of that released by the individual enzymes

## Discussion

To our knowledge, this is the first report on the gene cloning, expression, and characterization of a GH51 α-l-arabinofuranosidase (Ac-Abf51A) from the genus *Alicyclobacillus*. This genus contains unusual ω-alicyclic fatty acids in the cell membrane and is thus highly thermoacidophilic [27]. It produces a spectrum of GHs, including GH9 and GH51 cellulases [28–30], GH10 xylanase [31], GH52 xylosidase [32], and GH113 mannanase [33]. Analysis of the sequence alignment and structure modeling indicated that Ac-Abf51A consists of two modules: the catalytic module of the frequently encountered (β/α)_8_ barrel fold and a C-terminal jelly-roll module. The overall structure of Ac-Abf51A is very similar to that of the six previously published Abfs from bacteria. Furthermore, among the nine conserved residues within the GH51 arabinofuranosidases, the catalytic acid/base Glu175 is involved in the formation of a hydrogen bond with the conserved His244, and the Arg69 residue appears to keep the catalytic nucleophile Glu294 deprotonated [34]. All these data strongly support the conclusion that Ac-Abf51A is a putative GH51 Abf.

SignalP 4.1 analysis indicated the absence of putative Sec-type signal peptide in Ac-Abf51A. Prediction of the subcellular location using pSORTb (http://www.psort.org/) indicated that Ac-Abf51A is similar to AbfATK4 from *Geobacillus caldoxyloslyticus* TK4 [35] and AF from *Bacillus stearothermophilus* T-6 [36], showing a high probability in the cytoplasmic and extracellular space with the localization scores of 5.86 and 3.83, respectively. In contrast to most bacterial GH51 Abfs that are optimally active under neutral and mesophilic conditions [20–22], Ac-Abf51A was most active at pH 6.0 and 60 °C, and remained stable between pH 4.0 and 11.0, 65 °C for 12 h. However, compared to all characterized Abfs of GH 51, Ac-Abf51A showed distinct and broader substrate specificity [23, 24, 26]. Not surprisingly, the enzyme was most active on the artificial substrate 4-nitrophenyl-α-l-arabinofuranoside. Unlike GH3 and GH43 Abfs having both Abf/β-d-xylosidase activities, most GH51 Abfs have no ability to hydrolyze 4-nitrophenyl-β-d-xylopyranoside. However, Ac-Abf51A in this study, ArfB from *C. stercorarium* [18], and AF from *B. stearothermophilus* T-6 [36] have high Abf activity with weak β-xylosidase activity, which are less than 1 % relative to the Abf activities. The inactivity of Ac-Abf51A to gum arabic also indicates its specificity towards the furanosidic conformation and α-linkages. Ac-Afb51A did not display activity against red debranched arabinan, which is a substrate specific for the endo-1,5-arabinanase activity, indicating its inability to cleave the internal α-1,5-linkage. On the other hand, Ac-Afb51A yielded arabinose as the sole hydrolysis product from branched and debranched sugar beet arabinan. These results showed Ac-Afb51A was an exo-acting enzyme that had hydrolytic activity against α-1,2-, α-1,3-, or α-1,5-linked non-reducing terminal l-arabinofuranose residues [37, 38]. Moreover, it exhibited higher activity against branched (1.9-fold) than debranched sugar beet arabinan, suggesting it preference for the α-1,2- and α-1,3- rather than α-1,5-glycosidic bonds.

Surprisingly, Ac-Abf51A exhibited activity against water-soluble wheat arabinoxylan, releasing arabinose, xylobiose, xylotriose, and xylotetraose after a prolonged incubation. It has been reported that the GH51 Abf from *G. stearothermophilus* T-6 [19] can accommodate xylopyranosidic substrate in the active site. However, whether it has endo-xylanase activity was not determined. The time course analysis of wheat arabinoxylan hydrolysis revealed that Ac-Abf51A preferentially removes α-1,2- and α-1,3-linked arabinofuranose side chains from arabinoxylan, and hydrolyzes internal β-1,4-linkages at a much lower rate. Furthermore, Ac-Abf51A also had ability to hydrolyze xylohexaose, xylopentaose, and xylotetraose, but not xylotriose and xylobiose, to shorter xylooligosaccharides. The catalytic efficiency of Ac-Abf51A improved slightly with increasing polymerization degree of xylooligosaccharides up to six (the catalytic efficiency ratio of xylotetraose, xylopentaose, and xylohexaose was 1:5.8:16.4). These results suggested that Ac-Abf51A exhibits an endo-mode of action against xylan. The replacement of Glu175 or Glu294 with Gln resulted in a complete loss of both α-l-arabinofuranosidase and endo-xylanase activities, indicating that Ac-Abf51A catalyzes the hydrolysis of these substrates at the same active center. Thus, this is the first report on a novel GH51 Abf having endo-xylanase activity.

Synergy of hydrolytic enzymes has been extensively studied in prior studies [39–41], which is a useful way to improve sugar yield at low cost. When Ac-Abf51A was incubated in combination with endo-xylanase XynBE18 sequentially or simultaneously to degrade wheat arabinoxylan, more reducing sugars were released. There was no significant synergy increase in sequential reaction of Ac-Abf51A followed by XynBE18. But when Ac-Abf51A was added after XynBE18, the amounts of arabinose, xylobiose, and xylotriose released were higher than that by the two enzymes acting separately. The results suggest that efficient degradation of wheat arabinoxylan is achieved by the first cleavage of the main chains with XynBE18 followed by branch removal with Ac-Abf51A. Moreover, the synergy degree of the simultaneous reactions was higher than that of the sequential reactions. As results, Ac-Abf51A plays a role in the release of both arabinose and xylose moieties from arabinose-containing xylooligosaccharides generated by xylanase [42]. Discovery of this bifunctional Abf/xylanase is seminal to the structural analysis of the catalytic mechanisms of GH51 Abfs. Considering the distinct substrate specificity and biochemical stability, Ac-Abf51A has great application potentials for biomass degradation and refining.

## Conclusions

Thermoacidophilic *Alicyclobacillus* sp. A4 is an excellent producer of CAZymes, especially of hemicellulases and cellulases. This study firstly reported the gene cloning, expression and characterization of an auxiliary α-l-arabinofuranosidase of GH51. Neutral Ac-Abf51A remained stable over a broad pH range (pH 4.0–11.0) and high temperature (70 °C), and exhibited an exo-type mode of action towards polyarabinosides. It had ability to release arabinose, xylobiose, and xylotriose from wheat arabinoxylan, and exhibited endo-type action towards xylooligosaccharides with a lower level of activity. This is the first report of a GH51 Abf that has endo-xylanase activity. The broad substrate specificity, biochemical stability, and great synergistic effect with xylanase make Ac-Abf51A a potentially interesting enzyme for application in industrial biomass degradation.

## Methods

### Strains and culture medium

*Alicyclobacillus* sp. A4 CGMCC 3147 (whole genome sequenced, unpublished) was maintained in our laboratory [31]. *Escherichia coli* strains Trans I-T1 and BL21 (DE3) (TransGen) were maintained in Luria–Bertani (LB) broth or on agar plates at 37 °C for gene cloning and expression, respectively. Substrates were purchased from Sigma or Megazyme. All other chemicals were of analytical grade and commercially available.

### Gene cloning and sequence analysis

The gene fragment of *Ac*-*abf51A* (no signal peptide coding sequence based on SignalP 4.0 prediction) was amplified from the genome of strain A4 by PCR using the expression primers (abf51F: 5′-CGCCATATGTCCAAGTCGATTGCACGTATG-3′ and abf51R: 5′-GACGCGGCCGCCACGCGCAATCGAATGACG-3′, *Nde*I and *Not*I sites underlined, respectively). The PCR products were gel-purified with Agarose Gel DNA Purification kit (TaKaRa), ligated into a pGEMT easy vector (Promega), and transformed into *E. coli* Trans I-T1 for sequencing. The nucleotide sequence was assembled and analyzed using Vector NTI Advance 11.5 software (Invitrogen). BlastN and BlastP programs (http://www.ncbi.nlm.nih.gov/BLAST/) were used to analyze the nucleotide and deduced amino acid sequences, respectively.

### Protein expression and purification

*Ac*-*abf51A* was obtained by digesting the recombinant plasmid pGEMT-*Ac*-*abf51A* with restriction endonucleases *Nde*I and *Not*I (TaKaRa), and was cloned into the *Nde*I and *Not*I sites of pET-30a(+) vector (Novagen). The recombinant plasmid pET-*Ac*-*abf51A* was then transformed into the *E. coli* BL21 (DE3) competent cells. The positive transformants harboring pET-*Ac*-*abf51A* were identified by PCR amplification, and further cultured in LB medium supplemented with 100 μg/mL kanamycin at 37 °C to an A_600_ of approximately 0.6. Gene expression was induced at 30 °C for 6 h by isopropyl-β-d-1-thiogalactopyranoside (IPTG) with a final concentration of 0.6 mM. The induced cultures were centrifuged at 12,000×*g* for 5 min at 4 °C to harvest cells. The pellet (5 g) re-suspended in 25 ml of lysis buffer (20 mM Tris–HCl, 0.5 M NaCl, pH 7.6) was sonicated with an Ultrasonic Cell Disruptor (Scientz) on ice with 100 short bursts at 150 W of 6 s followed by intervals of 15 s for cooling. After removal of cell debris by centrifugation, the supernatant was subjected to Ni^2+^-NTA chromatography with a linear 20–300 mM imidazole gradient in 50 mM Tris–HCl, 500 mM NaCl, pH 7.6. The fractions exhibiting enzyme activities were pooled, concentrated, and assayed by sodium dodecyl sulfate–polyacrylamide gel electrophoresis (SDS-PAGE). The Bradford protein assay was employed to determine protein concentration at 595 nm with bovine serum albumin as the standard.

### Enzyme assay

Abf activity was assayed as described previously [21]. The standard reaction system consisted of 250 μL of appropriately diluted enzyme and 250 μL of McIlvaine buffer (pH 6.0) containing 2 mM 4-nitrophenyl-α-l-arabinofuranoside. After incubation at 60 °C for 10 min, 1.5 mL of 1.0 M Na_2_CO_3_ was added into the system to terminate the reaction. The amount of *p*-nitrophenol released was determined spectrophotometrically by reading the absorbance at 405 nm. One unit of Abf activity was defined as the amount of enzyme that released 1 μmol of 4-nitrophenol per min under the assay conditions.

The dinitrosalicylic acid (DNSA) method [43] was used to assay the xylanase activity. The reaction system consisted of 100 µL of appropriately diluted enzyme solution and 900 µL of 1 % (w/v) xylan in McIlvaine buffer (pH 6.0). The reaction mixture was incubated at 60 °C for 10 min, then 1.5 mL of DNSA reagent was added to terminate the reaction. The mixture was boiled for exactly 5 min and cooled down to room temperature. The color formation was monitored at 540 nm and quantified by comparison with a standard curve of d-xylose. One unit of xylanase activity was defined as the amount of enzyme releasing 1 μM of reducing sugars equivalent to d-xylose per minute under assay conditions. Each reaction was performed in triplicate, as was each experiment.

### Biochemical characterization

The optimal pH for the Abf activity of purified recombinant Ac-Abf51A (3 μg/mL) was determined at 37 °C for 10 min in 100 mM McIlvaine buffer (pH 3.0–8.0), 100 mM Tris–HCl (pH 8.0–9.0) and 100 mM glycine-NaOH (pH 9.0–10.0). The stability of Ac-Abf51A under different pH conditions was assessed by measuring the residual activities under standard conditions after incubation of the enzyme (60 μg/mL) in the buffers as described above at 37 °C for 1 h. The optimal temperature for Ac-Abf51A activity (3 μg/mL) was carried out at various temperatures from 20 to 80 °C in 100 mM McIlvaine buffer (pH 6.0). Thermal stability was determined by measuring the residual activities under standard conditions [100 mM McIlvaine buffer (pH 6.0), 60 °C for 10 min] after incubating the enzyme (60 μg/mL) at 60 °C, 65 °C, 70 °C, and 80 °C for different times without substrate.

The Abf activity of Ac-Abf51A was also analyzed in the presence of 5 mM of NaCl, KCl, MgSO_4_, CaCl_2_, AgNO_3_, ZnSO_4_, FeCl_3_, NiSO_4_, CuSO_4_, MnSO_4_, CrCl_3_, CoCl_2_, Pb(CH_3_COO)_2_, SDS, EDTA, and β-mercaptoethanol. The system without any additive was used as control.

### Substrate specificity and kinetic parameters

The Ac-Abf51A activities against different substrates were measured as described below. When using 1 mM of 4-nitrophenyl-glycosides including 4-nitrophenyl-α-l-arabinofuranoside, 4-nitrophenyl-β-d-xylopyranoside, 4-nitrophenyl-α-d-galactopyranoside, 2-nitrophenyl-β-d-galactopyranoside, 4-nitrophenyl-α-d-glucopyranoside, 4-nitrophenyl-α-l-arabinopyranoside, *p*-nitrophenyl-acetate, α-1,5-linked arabinooligosaccharides (arabinobiose, arabinotriose, and arabinotetraose), and 4-nitrophenyl-α-d-glucuronide as the substrates (Sigma), the enzyme activities were determined at pH 6.0, 60 °C for 10 min. When using 1 % (w/v) polysaccharides, such as sugar beet arabinan, debranched sugar beet arabinan, red debranched arabinan (dyed with Procion Red dye specific for the endo-1,5-arabinanase activity; Megazyme), gum arabic, water-soluble wheat arabinoxylan) and xylooligosaccharides (200 µg/mL of xylobiose, xylotriose, xylotetraose, xylopentaose, and xylohexaose; Megazyme) as the substrates, the enzyme activities were determined using high-performance anion exchange chromatography (HPAEC; Thermo Fisher Scientific) equipped with a Carbo-Pac PA200 column (3 µm × 250 mm). The reaction mixtures were incubated at pH 6.0, 37 °C for 12 h, followed by 10-min boiling water bath to terminate the reaction and centrifugation at 12,000×*g* for 5 min to remove unsolved residues. To remove the extra enzyme protein, the clear supernatants were centrifuged (10,000×*g*, 4 °C, 10 min) through a 3-kDa Amicon Ultra centrifugal filter (Millipore). The filtrates were diluted 100-fold in ddH_2_O, and 25 µl of each sample was subject to HPAEC analysis. NaOH (100 mM) was used to elute the saccharides at the flow rate of 0.3 mL/min. Arabinose, xylose, xylobiose, xylotriose, xylotetraose, xylopentaose, and xylohexaose served as standards.

Enzyme kinetic assays were performed at pH 6.0, 60 °C for 5 min with 4-nitrophenyl-α-l-arabinofuranoside (1–10 mM) as the substrate. The *K*_m_ and *V*_max_ values of Ac-Abf51A were plotted based on Lineweaver–Burk method. The catalytic efficiency (*k*_cat_/*K*_m_) of Ac-Abf51A towards xylooligosaccharides was determined following the Matsui equation as previously described by Cervera-Tison et al. [44].

### Mutant construction and characterization

To further verify whether the α-l-arabinofuranosidase and endo-xylanase activities of Ac-Abf51A were derived from the same module and the active center, truncation mutants of single module (Ac-Abf51An and Ac-Abf51Ac) and substitution mutants (Glu175 and Glu294) were performed using the Quik-Change method (Stratagene). All mutations were generated in the wild-type Ac-Abf51A DNA in the expression plasmid. The resulting mutant plasmids were transformed into *E. coli* BL21 (DE3) cells and confirmed by DNA sequencing. Expression, purification, and characterization of the mutant enzymes followed the same procedure as for the Ac-Abf51A.

### Synergetic effects of Ac-Abf51A and xylanase XynBE18

Endo-xylanase XynBE18 from *Paenibacillus* sp. E18 [41, 45] is an excellent xylooligosaccharide producer and was selected for enzyme combination. The reaction mixtures were composed of 900 μL of 0.5 % (w/v) water-soluble wheat arabinoxylan and 100 μl of enzyme(s) (0.5 U each of XynBE18 and/or Ac-Abf51A). All reactions were carried out at 37 °C in 100 mM McIlvaine buffer (pH 6.0). After 12-h incubation, the reaction mixtures were immersed in boiling water for 10 min to stop the reactions. Blank controls containing substrate alone were treated under the same conditions. For the sequential reactions, the second enzyme solutions were added after heat denaturation. The hydrolysates were assessed using HPAEC as described above. The degree of synergy was defined as the ratio of reducing sugar equivalents released when enzymes were incubated simultaneously or sequentially to the sum of the reducing sugar equivalents released by each enzyme alone.

## Additional files

10.1186/s13068-015-0366-0 The three-dimensional structure model of Ac-Abf51A, E175 and E294 are putative catalytic residues.
10.1186/s13068-015-0366-0 HPAEC analyses of the hydrolysis products of sugar beet arabinan (A) and debranched sugar beet arabinan (B). 1, the arabinose and xylooligosaccharide standards: Ara, arabinose; X1, xylose; X2, xylobiose; X3, xylotriose; X4, xylotetraose; X5, xylopentaose; and X6, xylohexaose. 2, the control of substrate incubated without enzyme for 12h; 3, the substrate hydrolysates by Ac-Abf51A treatment at pH 6.0 and 37°C for 12h.
10.1186/s13068-015-0366-0 HPAEC analyses of the hydrolysis products of xylooligosaccharides. (A) Xylotetraose. (B) Xylopentaose. (C) Xylohexaose. 1, the xylooligosaccharide substrates; 2, the xylooligosaccharide hydrolysates by Ac-Abf51A treatment at pH 6.0 and 37°C for 12h; 3, the xylooligosaccharide standards.

## Abbreviations

GH: glycosyl hydrolase
Abfs: α-l-arabinofuranosidases
LB: Luria–Bertani
IPTG: isopropyl-β-d-1-thiogalactopyranoside
Ac-Abf51A: α-l-arabinofuranosidase from *Alicyclobacillus* sp. A4
*K*_m_: Michaelis constant
*V*_max_: maximum reaction rate
*k*_cat_: turnover number
DNSA: 3,5-dinitrosalicylic acid
HPAEC: high-performance anion exchange chromatography
PDB: protein database
