# Supplementary figures and images for: A novel bifunctional GH51 exo-α-l-arabinofuranosidase/endo-xylanase from Alicyclobacillus sp. A4 with significant biomass-degrading capacity
Source: Biotechnol Biofuels. 2015 Nov 30;8:197. doi: 10.1186/s13068-015-0366-0 (PMC4666033; doi:10.1186/s13068-015-0366-0)

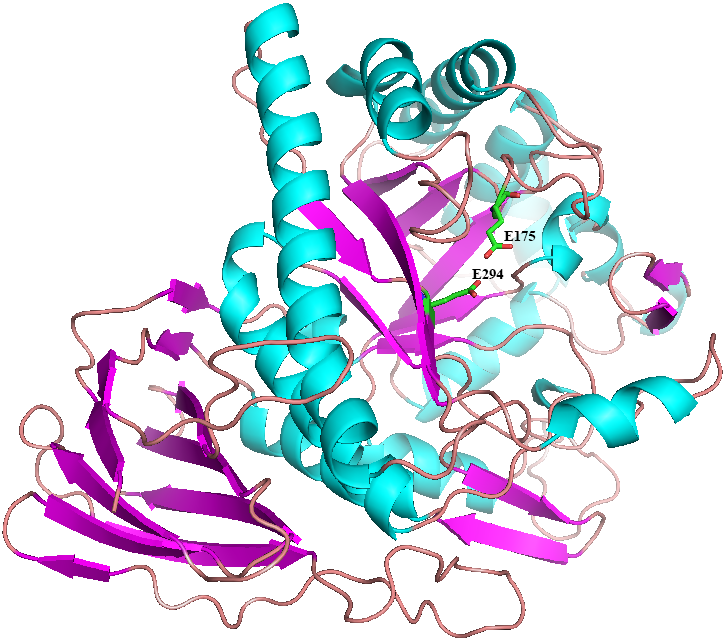

Supplement: Supplementary file 1 — 10.1186/s13068-015-0366-0 The three-dimensional structure model of Ac-Abf51A, E175 and E294 are putative catalytic residues. [file 13068_2015_366_MOESM1_ESM.tiff]

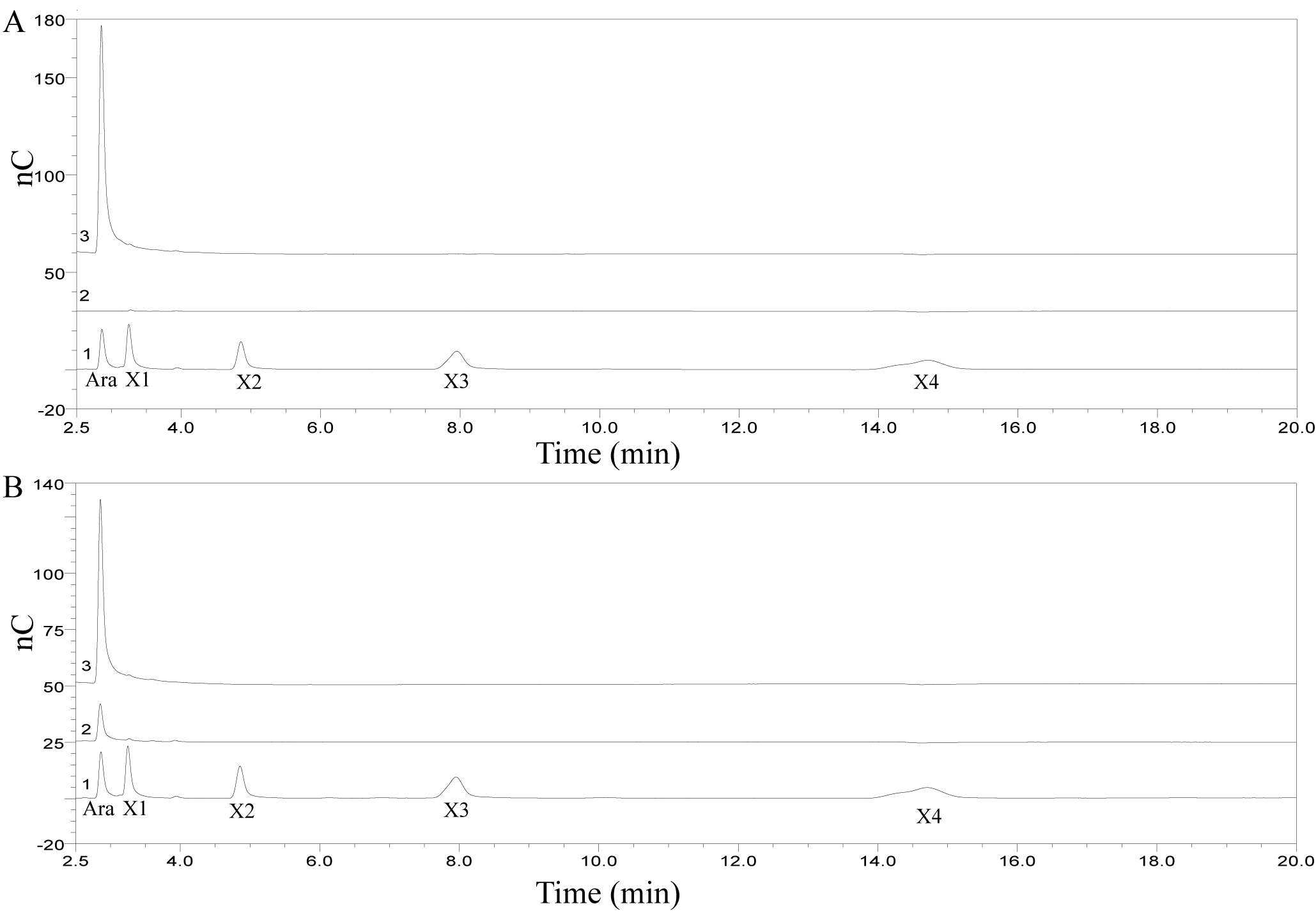

Supplement: Supplementary file 2 — 10.1186/s13068-015-0366-0 HPAEC analyses of the hydrolysis products of sugar beet arabinan (A) and debranched sugar beet arabinan (B). 1, the arabinose and xylooligosaccharide standards: Ara, arabinose; X1, xylose; X2, xylobiose; X3, xylotriose; X4, xylotetraose; X5, xylopentaose; and X6, xylohexaose. 2, the control of substrate incubated without enzyme for 12h; 3, the substrate hydrolysates by Ac-Abf51A treatment at pH 6.0 and 37°C for 12h. [file 13068_2015_366_MOESM2_ESM.tiff]

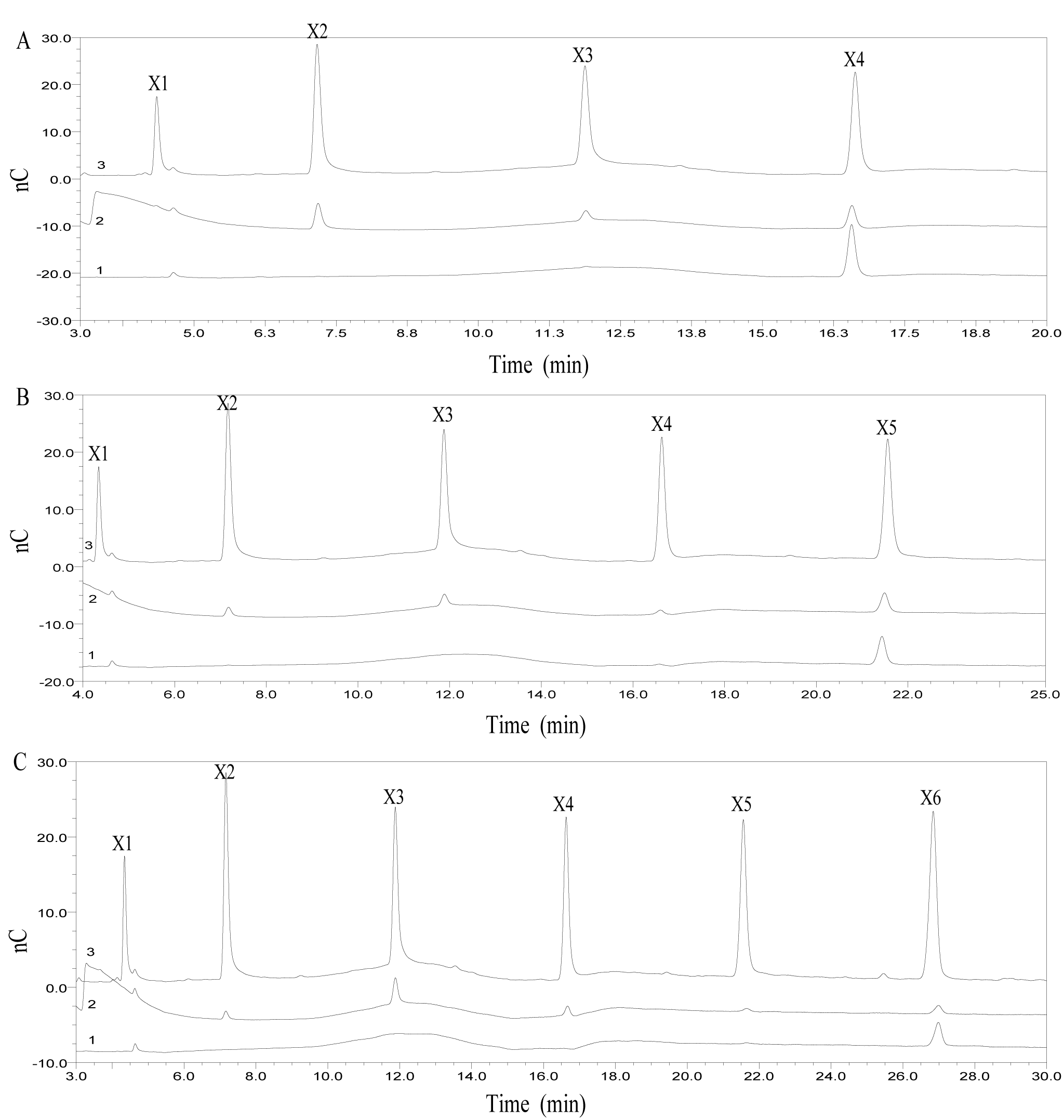

Supplement: Supplementary file 3 — 10.1186/s13068-015-0366-0 HPAEC analyses of the hydrolysis products of xylooligosaccharides. (A) Xylotetraose. (B) Xylopentaose. (C) Xylohexaose. 1, the xylooligosaccharide substrates; 2, the xylooligosaccharide hydrolysates by Ac-Abf51A treatment at pH 6.0 and 37°C for 12h; 3, the xylooligosaccharide standards. [file 13068_2015_366_MOESM3_ESM.tiff]
